# How Metaphors of Organizational Accidents and Their Graphical Representations Can Guide (or Bias) the Understanding and Analysis of Risks

**DOI:** 10.3390/jintelligence11100199

**Published:** 2023-10-12

**Authors:** Fabrizio Bracco, Martina Ivaldi

**Affiliations:** Department of Educational Sciences, University of Genova, 16121 Genova, Italy; fabrizio.bracco@unige.it

**Keywords:** organizational accidents, visual models, metaphors, risk perception, conceptualizing safety

## Abstract

The history of safety science has seen the flourishing of several models and metaphors aimed at describing organizational accidents’ dynamics. Metaphors and their graphical representations are powerful tools to frame risks and adverse events in socio-technical systems; they help in coping with systemic complexity but can also become a constraint and even bias the understanding of our environment. This paper aims to investigate how metaphors and their graphical representations influence the comprehension of organizational accidents, how they could be misinterpreted, and, as a result, generate misunderstandings of events. To address these questions, we analyze three paradigmatic accident causation models, typical of three phases in the evolution of models in the last century, describing how the related metaphors and depictions could influence the perception and understanding of risk factors. In addition, we present some possible misunderstandings that could be produced by the metaphor and graphical features of representations, with a particular focus on safety outcomes. Eventually, we provide a framework with the basic characteristics of an effective model and metaphor for the description and analysis of organizational accidents in modern complex socio-technical systems. This framework could be used as a guide for proposing new and more effective models in safety science.

## 1. Introduction

Metaphors can be potent instruments for influencing the way we think and look at our world. Metaphors encompass more than just rhetorical devices used for embellishment in speech, writing, or representations; they play a crucial role in perception and cognition (Eppler 2006; Van Hekken and Das 2019; Öztel and Hinz 2001). Metaphors involve the transfer of information from a familiar domain (referred to as the source domain, or the vehicle) to a new and relatively unfamiliar domain (known as the destination domain, or the topic) (Forceville 2006; Tsoukas 1991). By introducing new meanings and reframing available information, metaphors enable individuals to perceive phenomena or concepts from alternative perspectives. They prove useful for many purposes, for example, in simplifying complex ideas, such as explaining electricity as water flowing through pipes (Gentner and Gentner 1983).

Metaphors are conceptual phenomena through which humans comprehend and experience aspects of daily existence (Forceville 2006; Lakoff and Johnson 1980; Refaie 2003; Serig 2006). Consider the commonplace metaphor “time is money,” which conveys the idea that time is a valuable resource not to be squandered. This metaphor transcends a simple word comparison (“time” and “money”); it encapsulates the notion that time is a finite and precious commodity, susceptible to wastage, and deserving of prudent management. Metaphor goes beyond a mere matter of words; it is a conceptualization; “time” becomes a precious resource to safeguard, as it is the human mind that conceives it in such a manner (Lakoff and Johnson 1980).

As outlined by Coëgnarts and Kravanja (2014), metaphors possess two distinct levels: A conceptual dimension on the one hand, and a formal expression or manifestation level on the other. Since metaphors are thought processes, they can be expressed in a whole range of different forms, such as language, pictures, music, sounds, and gestures (Forceville 2006; Refaie 2003; Serig 2006). According to Forceville (2006), metaphors can be conveyed either exclusively within one mode (e.g., visual, verbal, aural) or span across multiple modes (e.g., audio-visual, verbal-visual, or audio-verbal-visual). In monomodal metaphors, target and source are primarily or entirely conveyed through a single mode, such as the linguistic expression “argument is a war” (Lakoff and Johnson 1980); on the other hand, in multimodal metaphors, target and source are each represented in different modes, such as visual and auditory, like a cat with an elephant snout emitting trumpeting sounds in an animated film (Forceville 2006).

Although different modes of communication may convey similar meanings, differences exist in their expressive capacities. For instance, while in language the chronological dimension of events is expressed by the use of syntax and adverbs (before, after), visual representations need to use spatial rather than temporal signals; for instance, depicting a past event as smaller or positioned behind a current event. These distinctions in manifestations can have significant implications for how individuals conceptualize various phenomena (Refaie 2003).

The field of industrial safety flourishes with metaphors that conceive organizational phenomena, such as accidents, into tangible terms. Particularly, metaphors have been used to explain how accidents occur since the beginning of the research field (Le Coze 2019). For instance, one common metaphor for accidents describes organizational failures as a cascade of falling dominoes; each domino represents an organizational factor dependent on the others. This metaphor serves to illustrate how accidents stem from a series of interconnected causes and has often been accompanied in literature by a graphical representation (Heinrich 1941). Metaphors in safety are useful for conveying a particularly complex idea, such as the development of accidents. By framing organizational failures in concrete terms, a metaphor and its graphical representation can effectively communicate abstract or complex concepts in a more accessible way. From a cognitive standpoint, they can significantly influence how individuals interpret the corresponding phenomena (Le Coze 2019).

The use of metaphors and their graphical representations for explaining accidents may also possess several potential drawbacks. For instance, metaphors might overemphasize similarities between entities that are fundamentally distinct: Metaphors assert that A has some properties in common with B (e.g., accident development such as falling dominoes); however, A is not B (in organizations, there are not dominoes, and they cannot be pushed in one direction or be physically stopped) (Eppler 2006). The juxtaposition of two distinct aspects (“accidents are like dominoes’’) draws attention to the dominant features embodied by the metaphor, encouraging people to see accidents in terms of sequential and linear characteristics; simultaneously, it diverts attention from traits that do not align with this concept, such as the simultaneous occurrence of events or redundancy. Conceptualizing a phenomenon through metaphorical lenses accentuates specific features while relegating others to the background. This runs the risk of fostering a selective and incomplete perspective of the accident, leading to a biased understanding (Barner 2008). 

Furthermore, even the adoption of a specific graphical representation of that metaphor has the effect of guiding the observers’ minds in framing a domain of knowledge accordingly (Refaie 2003). Each graphical representation of the metaphor is a map representing a landscape from a specific point of view: It highlights some aspects, leaves other aspects in the background, and completely neglects others (Petridis and Chilton 2019; Ziemkiewicz and Kosara 2008). Moreover, graphical representations can possess varying degrees of implicitness, and the construction of meaning is jointly negotiated by creators and observers; images extend beyond the simple depiction of real events. This means that associative interpretations can significantly vary among individuals (Refaie 2003). 

It is therefore important to understand how the phenomenal experience of metaphors and their depictions may guide cognition, facilitate some processes, sometimes even bias them, and could also be the ground for misunderstandings and wrong assumptions (Morgan 2011). 

Given the relevance of metaphors in representing and processing complex data, in this paper we will focus on the use of metaphors as models adopted to represent what Weick (1987) defined as a “dynamic non-event”: Safety in complex socio-technical systems. This paper aims to investigate the influence of metaphors and their graphical expressions on an understanding of organizational risks and accidents. Specifically, we question:

1. How do metaphors influence the comprehension of organizational accidents?
2. How can the graphical representation of metaphors shape the observers’ comprehension of accidents?

To address these questions, we will choose three accident models paradigmatic of the main eras in safety science and analyze them in terms of the effectiveness of their related metaphors and their visual representations. We will describe how the related metaphors and graphical representations may influence the understanding of risk factors. Our investigation will encompass two levels of analysis. First, we will delve into the influence of metaphorical concepts on the comprehension of accidents. Second, we will place a focus on the comprehension of accidents through the utilization of formal graphical expressions of metaphors. 

In the subsequent sections, we will initially present a classification and development of accident models from the safety science literature. In the following sections, we will present each accident metaphor and its graphical representation, focusing on (1) how people might conceptualize accidents through the first and second models, and (2) how the visual characteristics of metaphorical expressions can shape the comprehension of organizational failures through the second and third models. In each part, we will also focus on some possible misunderstandings that could be produced by the metaphor and their visual expressions, with a particular emphasis on safety outcomes.

Eventually, this paper will provide a framework with the basic characteristics of an effective model and metaphor for the description and analysis of organizational accidents. This framework could be used as a guide for proposing new and more effective models in safety science.

## 2. Accident Metaphors

Current sociotechnical systems are characterized by complex interactions between operators, teams, technology, the organization, and their physical and social environment (Reason 1997). They pose an interesting challenge to those involved in their design and management, but also to safety scientists that try to investigate and model the complex dynamics occurring in these organizations (Hollnagel 2012; Nuutinen and Norros 2009). Safe operations are therefore based on the optimal design and management of the several factors at play (Woods et al. 2010). Metaphors have been employed to address fundamental challenges in understanding the complexity of sociotechnical systems and conceptualizing safety. In an effort to visually capture various facets of complex system functioning, visual representations of metaphors strive to enhance understanding of organizational events. According to Le Coze (2019), accident metaphors have played a significant role in establishing safety as an independent field of research, distinct from other disciplines. As these metaphors have often been conveyed from consultants to professionals or from managers to operators, they can permeate the very fabric of an organization and become embedded in its cultural context. These metaphors can be observed in various aspects, including the investigation processes, the organizational improvement actions, and the design of training programs (Swuste et al. 2014). 

As stated by Hovden et al. (2010, p. 955), “accident models affect the way people think about safety, how they identify and analyze risk factors and how they measure performance. Accident models can be used in both reactive and proactive safety management”. Thus, the question of how these metaphors affect the perceptions of workers, managers, and practitioners regarding organizational failures seems to be a central aspect to consider. 

Safety science literature provides several reviews about the classification and development of accident models, and they generally represent it as an evolution from linear to complex models (Hollnagel 2004, 2009; Hovden et al. 2010; Lundberg et al. 2009; Pillay 2015; Roelen et al. 2011; Saleh et al. 2010; Fu et al. 2020). Hollnagel (2004) frames this trend from linear, or sequential models, to complex linear models, to complex non-linear models. In a more nuanced framing, Pillay (2015) claims that our understanding of accident dynamics and prevention has followed several ages, starting from the focus on technology (with a linear approach to events analysis), to the resilience approach (capable of representing complex non-linear dynamics).

The models have been proposed according to the technical and organizational nature of the system they aim to represent. This means that there is not an ideal or all-purpose model since the systems are different. Moreover, each model would be more suitable for the specific kind of sociotechnical system it aims to represent; for instance, a model suitable for the representation of accidents in manufacturing could be ineffective or even misleading for the healthcare system (Fu et al. 2020; Hollnagel 2008, 2009). In general, linear models could be suitable for understanding and investigating accidents in linear systems. As the complexity and interconnectedness of a system’s elements increase, linear-systemic models may be suitable, and for even more complex systems, non-linear models could tackle the accident dynamics. However, non-linear models could not be suitable to represent sequential dynamics if they are more focused on qualitatively describing the interconnections among elements and not the temporal development of the accident (Hollnagel 2008).

Although there are many models and metaphors present in safety science, in this paper we will focus on three models that are representative of the three main phases of the evolution of safety paradigms and are based on a metaphor (Hollnagel 2008, 2009). We will apply the above-mentioned research questions to the three models, namely: how does the metaphor and/or its visual representation guide and bias the comprehension of organizational accidents?

The evolution of accident models followed the technological and organizational development of productive systems, going from simple and linear systems (e.g., the assembly line) to complex and non-linear systems (e.g., healthcare). The three phases are (Pillay 2015):

1. Simple sequential linear accident models: The basic linear models operate under the assumption that accidents occur as a result of a chain of interconnected events or circumstances that occur in a sequential and straightforward manner. Therefore, by removing one of the causes in this linear chain, accidents can be prevented.
2. Complex linear models: They operate under the assumption that accidents arise from a blend of hazardous conditions within the system and unsafe actions. These factors align along a linear trajectory, with those farther from the accident associated with organizational or environmental actions and those closer to the accident tied to human interactions. The underlying belief is that accidents can be averted by reinforcing barriers and defenses.
3. Complex non-linear models: their fundamental assumption states that accidents could be best understood as outcomes of multiple variables that interact with one another in real-world settings; only by comprehending the combination and interaction of these various factors can accidents be genuinely understood and effectively prevented.

To address the two research questions outlined above, we will apply them to three accident models, paradigmatic of the three phases described by Pillay (2015). Namely, the domino model for the linear approach (Heinrich (1941), the Swiss cheese model for the complex linear approach (Reason 1990), and the functional resonance analysis method (FRAM) for the complex non-linear approach (Hollnagel 2012). We choose these models not only because they are representative of the main milestones in the evolution of safety models but also because they are suitable cases to answer the question of whether the choice of a metaphor and/or its graphical representation can affect the way people think about safety phenomena. As argued before, metaphors can be examined at the conceptual level and at the graphical representation level. In particular, we will argue that the domino metaphor might affect cognition about safety issues mainly because of its conceptual nature, while we believe its graphical representation is not relevant to account for potential cognitive misrepresentations. This model will be analyzed to answer question 1: how do metaphors influence the comprehension of organizational accidents?

To analyze an example of a combination concerning question 1 and question 2 (how do metaphors influence the comprehension of organizational accidents? And how can the graphical representation of metaphors shape the observers’ comprehension of accidents?), we will show how the Swiss cheese model could foster ineffective cognitive representations of organizational accidents both for the metaphor per se and the visual instantiation with which the model has been communicated. Eventually, as a problem concerning question 2, we will see how the FRAM metaphor is very appropriate and reliable in the description of organizational accidents, but its visual rendering could be ineffective in favoring the cognitive representation of the accident dynamics. 

### 2.1. Linear Models: The Domino Metaphor

**Figure 1:**
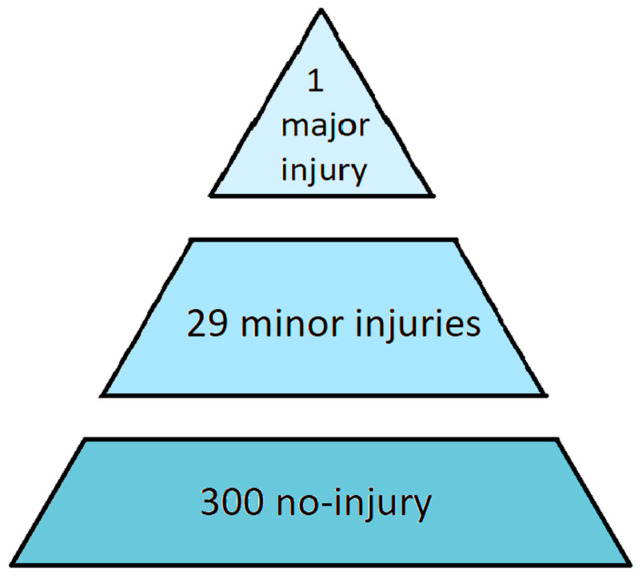
Heinrich’s (1941) pyramid. The graphical representation of statistical relationships between major injuries, minor injuries, and no-injury.

Sequential models have been developed to explain accidents as a series of events, offering tools for managers and researchers to identify factors leading to failures (Woods et al. 2010; Swuste et al. 2014). One of the earliest models to elucidate the precursors of accidents is Heinrich’s pyramid. Heinrich (1941) proposed that accidents occur as a result of preceding events, such as near misses or unsafe acts. These events are metaphorically represented as layers of a pyramid, with the actual injury at the pinnacle. Heinrich’s original work in the 1930s aimed to establish statistical relationships between major injuries and other critical safety events, based on data from the insurance industry. According to Heinrich (1941), the ratios of near misses (i.e., no-injury accidents), minor injuries, and major injuries would result in a triangular shape (300:29:1) (Figure 1). 

In other words, out of 330 events, 300 would not result in any injury (at the bottom of the pyramid), 29 would lead to minor injuries (middle layer), and one would result in a major injury (at the top of the pyramid). Over time, Heinrich’s pyramid has been modified by various authors. In the book “Practical loss control leadership”, (Bird et al. 1990) presented a pyramid with four layers: the base included 600 unsafe acts, followed by 30 near misses, 10 minor injuries, and finally, at the top, one major injury.

Heinrich’s pyramid has faced criticism regarding the statistical relationships identified by the author (Marsden 2017; Yorio and Moore 2018). One of the challenges was the difficulty in retrieving minor injuries data or near misses using the reporting systems available at that time. Additionally, there is uncertainty about whether the same relationships hold true in all types of work contexts. Despite these criticisms, the model continues to be widely used by managers and is included in training materials for workers across various companies (Dekker 2019).

Studies on accidents and unsafe acts allowed Heinrich (1941) to develop another metaphor in an attempt to represent the causal link between the bottom of the pyramid (unsafe acts) and the top (major injury): a set of dominoes that toppled in a chain reaction.

**Figure 2:**
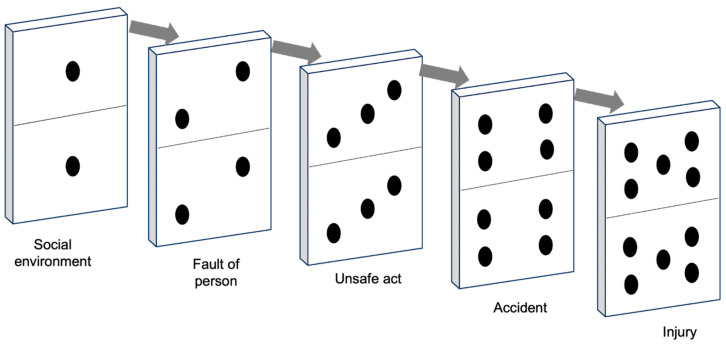
The domino model. A failure in a piece can compromise its stability and make it fall towards the following piece, activating a chain of causes and effects that ends in the adverse event.

The domino model was the first accident analysis technique used for prevention (Swuste et al. 2014). It illustrates the sequence of accidents from the initial cause (the first domino) to the actual failure (the latest domino) (Marsden 2017). When the first domino falls, it triggers the fall of the second, then of the third, and so on, with the injury being the final domino to fall. In Heinrich’s graphical representation, each tile represents an organizational element (Figure 2).

The farthest domino from the incident represents the individual’s ancestry and social environment. These factors can contribute to undesirable character traits, such as stubbornness or a violent temper, or may restrict educational opportunities. Both inheritance and environment lead to personal faults, which are represented by the second domino. The presence of certain personal traits, whether inherent or learned, provides immediate explanations for engaging in unsafe actions. According to Heinrich, these personal characteristics contribute to unsafe performance, such as standing under suspended loads or removing safeguards, represented by the third domino. Unsafe acts, in turn, lead to accidents such as objects falling from heights or individuals getting crushed between machine components, depicted as the fourth domino. These accidents directly result in fractures, lacerations, and other injuries, which are the latest domino.

Heinrich viewed the occurrence of an injury as the culmination of a sequence of events. Injuries depend on an interconnected chain of factors, where each element relies on the others. Removing any of the multiple components forming this chain prevents the injury from occurring (Marsden 2017). Heinrich (1941) argued that various factors can break the chain of events. Supervision and management play a crucial role in controlling employee actions and preventing unsafe practices. In the domino model, the unsafe acts of workers are key factors in accident prevention. 

According to Heinrich, prevention should focus on eliminating one domino. By breaking the chain of events, the latest tile (representing a potential accident) can be prevented from falling. Through this metaphor, Heinrich establishes a connection between preventive actions and accidents, providing managers with a theoretical framework for navigating the complexity of the system.

The metaphor suggests interpreting accident dynamics according to a fixed order, as in dominoes, where the direction of fall is unidirectional (Woods et al. 2010). This feature allows users of the model to reconstruct accidents back to the root cause, tracing from the last fallen domino to the underlying cause (Dekker et al. 2011).

From this metaphor, various accident design tools have emerged, including causal trees, event trees, and cause–consequence graphs. These graphical representations describe the linear causal flow of accidents and have been the primary tools in risk management within organizations for several decades. They have helped managers improve their understanding of organizational accidents (Hollnagel 2012; Pasman et al. 2018; Svedung and Rasmussen 2002).

Heinrich is recognized as a pioneer in theories of accident causation, particularly for his formulation of the theory of accident causation (Marsden 2017). However, these models have been criticized for being overly simplistic and failing to account for the complexity of socio-technical systems (Dekker 2002). Despite the criticisms, the domino model continues to be used today.

#### How Does the Domino Metaphor Guide (and Bias) the Comprehension of Organizational Accidents?

The use of the domino metaphor to illustrate a sequence of interconnected failures is not confined solely to the domain of industrial safety; it finds application among scholars and professionals across diverse fields, including the study of natural disasters, such as earthquakes (Pescaroli and Alexander 2015). This metaphor, employing falling tiles one by one, strives to elucidate how organizational accidents result from a series of interconnected causes (Marsden 2017).

The spatial dimension inherent to the domino metaphor, symbolized by the dominoes arranged consecutively, begets a temporal dimension in the conceptualization of events: The linear alignment of tiles mirrors the chronological sequence of the accident. This transformation of a spatial attribute (dominoes in a row) into a temporal characteristic of accidents molds the understanding, framing events in a sequential manner (first one cause occurs, followed by another).

Moreover, the spatial attribute within the domino metaphor carries an additional meaning: The interdependence of causes. It is not merely a matter of aligning dominoes; they must be positioned closely enough to make contact when the preceding tile falls. Conceptually, this signifies that the causes (the dominoes) rely on one another; the accident cannot occur unless the preceding causes are interconnected, much like the final domino cannot fall unless the preceding ones have toppled (Pescaroli and Alexander 2015).

According to Hollnagel (2012), establishing causal connections between events not only enables the prediction of future scenarios but also facilitates a retrospective analysis. Since failures occur in a sequence where the accident is only the final event (the latest domino), it is possible to trace back this sequence to find the cause. This retrospective analysis enables investigators to mentally reverse time, starting from the latest event and tracing back to the initial point (Dekker et al. 2011).

The domino metaphor has been valuable as an accident model that facilitates the analysis of organizational failures. It has provided managers with a simplified framework to comprehend the complexity of accident precursors and understand the root causes of accidents (Hudson 2014; Woods et al. 2010). Moreover, its popularity stems from its potential to disrupt the sequence of accidents by addressing the underlying causal factors (removing a domino). This has offered a practical way to link accident analysis with proactive measures aimed at preventing similar accidents in the future (Marsden 2017).

Notwithstanding these advantages of the model, its metaphor possesses some drawbacks when used to understand organizational accidents, making it susceptible to significant misinterpretations. While the model aids investigators in grappling with complexity during their analysis, the inherent features of the metaphor can result in an altered and misleading perspective of the accident. 

Two significant drawbacks of the metaphor are due to its sequential nature: Misattribution of causation, and oversimplification. Firstly, the domino metaphor, in addition to illustrating causal relationships, highlights the gradual compounding of failures, with each component falling as the result of a previous fall. This metaphor supports the idea that events unfold gradually, where one event leads to another (Dekker 2005; Hollnagel 2002). Consequently, the metaphor may suggest that events can be linked together only if one event directly precedes the other (Hollnagel 2012). For instance, if a nurse treats a patient, and that patient has an adverse reaction, the operator’s actions may be identified as the antecedent domino. This trend is typical of the fallacy of post hoc ergo propter hoc, which involves misattributing the cause of an event that precedes another and considering the subsequent event as the effect (Woods and Walton 1977). 

Secondly, it limits the consideration of multiple simultaneous causes that may have contributed to the accident. In the case of a medical error, factors such as similar packaging of medications (Bryan et al. 2021), patient bed switching, lack of coordination, stress, or other organizational conditions could have induced the error (Di Simone et al. 2020; Hoff et al. 2004). While the domino model allows investigators to explain the events preceding the accident with the fall of each tile, it becomes challenging to understand other organizational or contextual aspects that may have played a role (Dekker 2002). The linear causal nature of the domino model implies interpreting accidents as the outcome of a series of clearly identifiable events. In the metaphor, dominoes represent tangible physical objects with the power to change the system’s state by toppling the next tile. Consequently, accidents can be analyzed by breaking down the event into its individual components (the dominoes) (Chen et al. 2020). This decomposition enables investigators to bring order to complexity and infer which dominoes should be removed to prevent future failures (Hollnagel 2012). However, the metaphor runs the risk of promoting a mechanistic and reductionist interpretation of accidents. According to this approach, the functioning or malfunctioning of a system is explained by the functioning or malfunctioning of its constituent components, from a reductionist perspective, and the relationship among the parts is predictable and rigid. This can lead to a symmetry bias (Dekker et al. 2011), wherein cause and effect are often perceived as symmetrical (i.e., big effects should have had big causes). For instance, if an operator’s actions lead to an accident, the behavior is often considered highly negative because a serious consequence must have been caused by unacceptable conduct such as reckless decisions and unprofessional behavior. The issue with symmetry bias lies in its failure to account for all the systemic aspects contributing to an accident. When an operator makes a mistake in a specific context, it does not necessarily result in an accident. However, if the same mistake is made under different circumstances, it can indeed lead to harm (Gao and Dekker 2016).

Besides these drawbacks of the metaphor, there are also risks associated with the improper use of the model, which goes beyond its originally intended descriptive purpose and delves into post hoc interpretations of the phenomenon. The primary objective of the model is to identify the interconnected variables contributing to an accident. However, when investigators engage in retrospective analysis of the event’s causes, they may fall prey to the distortion induced by hindsight biases. Consequently, this analysis may incline toward biased interpretations of what occurred rather than offering a completely impartial, objective depiction of the phenomenon (Dekker 2002). Hindsight bias refers to the tendency to evaluate an event as more predictable, probable, and inevitable after it has occurred than before its occurrence (Fischhoff 1975). Consequently, the outcome seems to be the natural consequence of preceding events (Dekker 2003). Such misinterpretations frequently arise because possessing knowledge of the precise outcome tends to diminish people’s capacity to envision various alternative scenarios that could have led to that particular result. Conversely, awareness of the outcome prompts individuals to identify the sequence of causes, beginning from the initial state and culminating in the final state, portraying this causal chain as the sole plausible explanation for the result (Hudson 2014). This phenomenon has particularly negative implications for the comprehension of human behavior, encompassing errors and violations. In linear post-event descriptions, people’s actions may appear unreasonable, triggering questions about why the operators did not realize that their choices would lead to the accident (Brazier 2018). The problem with this interpretation is that it is easy to make predictions about what might have happened in hindsight, whereas the individuals experiencing the events could not have known the outcome of their actions in advance. In reality, within that specific context and circumstances, it is likely that their behavior followed a logical course; every action has its underlying rationale, history, and context. The reconstruction of events in linear sequences can often be far from the perspective of the operators involved (Dekker 2003). Operators may not have immediately understood what was happening, for instance, because they did not hear an alarm or were unable to intervene in time due to the rapid escalation of the situation. Investigators, as external observers, do not experience these same conditions. They analyze the situation by considering multiple viewpoints and gathering information from the environment. This simplifies the real conditions of uncertainty and ambiguity in which operators work (Hollnagel 2012). In reality, within complex systems, variables are so interdependent that even a slight variation in the system can have substantial consequences. Accidents can occur even when operators are performing their routine actions, which they typically do. In some cases, these actions might lead to failures, while in others, they might lead to success, depending on the specific situations and conditions (Dekker et al. 2008). As a result, the application of the linear model exposes investigators to the potential pitfall of interpreting events through the lens of hindsight. This, in turn, poses the risk of oversimplifying the complexity of the accidents, thereby overlooking the systemic and organizational factors that might have played a role in the error or violation (Hollnagel and Woods 2017). Indeed, the domino model encourages the pursuit of causes characterized by a direct cause-and-effect relationship; as previously emphasized, the tiles touching each other in this metaphor symbolize the interdependency of causes. The retrospective quest for the causal sequence ceases when no further backward linear relationships are identified, meaning when an active cause that can elucidate the activation of the sequence is absent. From this standpoint, anything lacking a direct cause-and-effect relationship is excluded from the model. Contextual factors that might contribute to errors and violations are often omitted from the sequence because they do not constitute directly observable causes of the accident. For instance, in the case of an inadvertent mix-up of two medications, it is more common to attribute the active cause to a nurse’s inattention or intentional violation rather than to a contextual factor such as the resemblance in packaging. This contextual aspect, associated with issues in the organization of medication management, does not qualify as an active cause; rather, it represents a risk condition that manifests only in specific circumstances (such as when the nurse is rushed or under stress). As a result, the domino model can lead to the attribution of accident-triggering causes to human actions, as workers’ behavior constitutes an active cause and is immediately observable within the sequence, in contrast to other latent systemic conditions that interact with various risk factors.

The domino metaphor, with its linear characteristics, is at risk of being applied rigidly to the complex and dynamic context of safety, leading to two potential organizational threats: Blaming workers for organizational accidents and orienting improvement actions only towards operators. Firstly, an excessive allocation of accountability to individuals involved in accidents represents a significant risk as it perpetuates a blame culture within organizations, where the primary focus is on finding a responsible party whenever an accident occurs (Dekker et al. 2011). This trend is well documented in numerous accident reports. An analysis conducted by Holden (2009) on 27 major aviation accidents revealed that in 26 out of 27 cases (96%), pilots or flight crew were deemed to be the main causes of the failures. This approach to interpreting organizational failures, as described by Reason (2000), is referred to as the person approach. It assumes that errors or violations primarily stem from factors such as inattention, lack of motivation, negligence, or imprudence, thereby attributing the causes of accidents mainly to individuals. The blame approach is highly detrimental to systems, as it leads to analyses that pay little attention to organizational factors and overly focus on individuals. This poses a significant risk as it diverts the investigation’s primary objective, which is to understand the causes of events, towards a search for a culprit. The main motivation behind analyzing an event should be to comprehend the underlying dynamics, not to identify and punish someone, following the logic of eliminating “bad apples” (Dekker 2017). Placing blame restricts learning about the system as it prevents the identification of systemic issues that could contribute to an accident (Catino 2008). 

Secondly, since the model tends to identify primary causes of accidents in humans, it may lead managers to focus preventive interventions solely on frontline workers, overlooking underlying risky organizational conditions, such as conflicting or impractical procedures for specific situations, organizational pressures to expedite work, or miscommunication between managers and operators (Hollnagel 2021). Failure to address these aspects puts the system at risk of accidents (Reason 1997). 

### 2.2. Complex Linear Models: The Swiss Cheese Model

**Figure 3:**
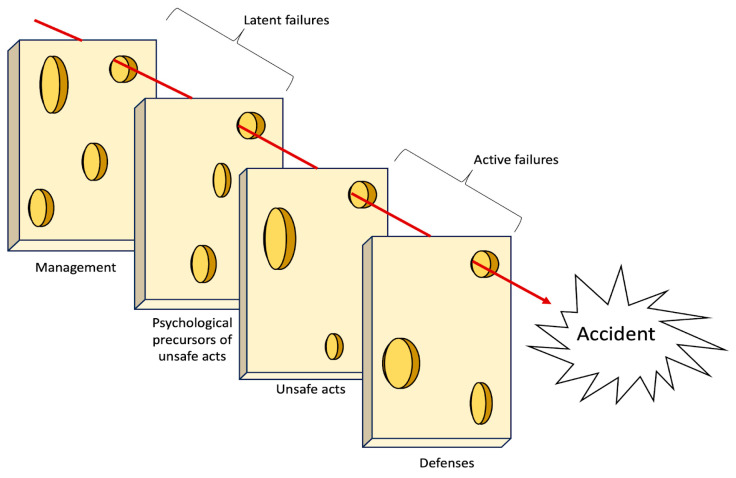
A generic representation of the Swiss cheese model.

The Swiss cheese model is an evolution from linear accident models, as it offers an explanation for accidents as a concatenation of multiple failures (Reason 1990). It shifts the focus from a single causal factor to a broader set of factors, including managerial aspects. The model recognizes that accidents are the result of a complex interplay of various factors, both active and latent, that can originate from different levels of the organization (Reason 1997). Reason employs this metaphor to explain the dynamics of organizational accidents. According to this model, organizations are equipped with defensive barriers. Due to the inherent complexity of the real world, these barriers are not flawless and may possess weaknesses. Reason classifies these weaknesses into two categories: Latent failures and active failures. Latent failures encompass all managerial aspects that may harbor critical issues, such as poor management decisions or flawed technological design. On the other hand, active failures refer to errors or violations committed by frontline workers. In the graphical representation, the safety barriers are depicted as slices of cheese, while the active and latent failures are represented as holes in these slices, which is why the metaphor is referred to as the “Swiss cheese model” (Figure 3).

The outline of the slices has been changed over time and has increasingly taken on a defined physical form; compared to the first version of the metaphor (Reason 1990), in which the barriers were simple two-dimensional rectangles, in 1997 (Reason 1997) they took on a three-dimensionality through the shading in the holes and in the outlines, and then became distinguishable slices of cheese in 2000 (Reason 2000). The 2000 version of the model was aimed at a medical audience. Recognizing that the medical target audience had less familiarity with human factors than other publics such as aviation (Larouzée and Le Coze 2020), Reason introduced a better graphical version of the model that closely resembled Swiss cheese, with increased thickness.

The number and labeling of the slices have changed over the years, and it is possible to find many instances of the same model; however, the basic elements are recurrent. Proceeding backwards along the timeline ending in the adverse event, immediately before the accident there is the slice representing the physical and procedural defenses (e.g., procedures, personal protective equipment, safety lockout, etc.). Before that, there is the slice of unsafe acts produced by operators (e.g., an error or a violation in a procedure). These acts may be performed because of the influence of the preceding slice, which represents the psychological preconditions for errors and violations (e.g., high workload, poor training, toxic leadership, etc.). The preconditions are favored by the last slice, which represents the organizational and management level, where bad decisions could be made, poor rules could be designed, and deviant organizational cultures could be fostered.

Typically, the presence of holes in the slices does not lead to an accident, as they are usually misaligned. However, an accident can occur when all the holes align across the slices, thus forming a trajectory for the mishap; that is, when all the organizational and individual factors interact and contribute to the outcome. It is a step further with respect to the Domino model since the holes represent potential failures and dormant conditions that might interact in the future and have negative outcomes. The binary representation of the domino’s tile (either upright, i.e., fully functioning aspect, or fallen, i.e., broken or malfunctioning aspect) is overtaken by the concept of holes, underperforming or risky conditions, that, in order to contribute to the accident, need to align and interact in a specific time and place. The holes in the slices closest to the event symbolize the active failures, as they represent the unsafe acts of the workers or equipment failures. On the other hand, the holes in the slices furthest from the event represent the latent conditions, which encompass organizational and managerial issues that exist upstream and that have created the conditions for the development of the risk into an accident. This powerful graphic representation (i.e., a solid matter characterized by scattered holes) aims to convey the concept of the simultaneous interaction of organizational, contextual, and individual factors (Larouzée and Le Coze 2020).

#### 2.2.1. How Does the Swiss Cheese Metaphor Guide (and Bias) the Comprehension of Organizational Accidents?

The Swiss cheese metaphor is used to provide an explanation of organizational failures and weaknesses inherently present in any sociotechnical system. According to the metaphor, organizational failures have a history that can span a considerable time frame and are not solely attributed to unsafe actions by workers (Reason 1990). 

We believe that the Swiss cheese model’s metaphor has several issues concerning both its metaphor per se and its visual representation. From a metaphorical standpoint, the conceptualization of accident dynamics is based on several metaphorical features that play a role in shaping the understanding of the accident. One of the most effective insights of the model is the notion of “latent failures”, distinct from those of “active failures”, but all symbolized as holes in the slices of cheese. The metaphor helps people to understand that in case of an accident, focusing on the nearest hole (the human) is not enough, and it is better to trace back along the chain of latent factors. This is why, for Reason, intervening on the individual is limited: It would change the specific slice, but if the holes at other levels persist, someone else will be destined to repeat the same errors. In this case, we are dealing with organizational accidents (Reason 1997), that is, mishaps in which the factors that generate the adverse event are to be sought in the latent conditions within the system and not just in human behavior. 

The holes in the metaphor are entities with their own ontological consistency, representing erosions of healthy parts of the system. Consequently, they epitomize imperfection as an inherent and even typical attribute of the system. It is worth noting that holes do not inherently signify threats; rather, it is their arrangement (the alignment) that dictates the occurrence of an accident (Reason 2000).

The Reason model has been widely adopted as a standard in safety studies and practices. It has contributed to accident investigation and, most importantly, has enabled the design of preventive interventions. However, the model has not escaped criticism (Reason et al. 2006). Some have noted its superficiality in the metaphor of slices and holes, arguing that it may be effective as a broad concept but is limited for practical application to real accidents (Luxhøj and Kauffeld 2003; Luxhøj and Maurino 2001; Shappell and Wiegmann 2000). Additionally, the model has been criticized for being rigid and linear (Dekker 2011).

For instance, the vision of holes in safety barriers is based on the dichotomy of normal and pathological, right and wrong. According to this model, there are no “good” holes, but they represent dangerous elements in the system. For instance, a violation should be described as a hole since it is a deviation from a procedure (safety barrier). However, sometimes violations are produced to avoid a bad outcome or are the necessary adaptation to the contingent situation; violations can contribute to system flexibility and enhance safety rather than compromise it. A notable example is the case of US Airways Flight 1549, which experienced bird strikes shortly after takeoff, leading to the loss of both engines. The crew quickly decided that the best action was an emergency landing in the Hudson River, despite the tower’s instructions suggesting a return to LaGuardia. This violation proved effective and saved the lives of all passengers and crew; on the other hand, following the procedure would have resulted in a fatal crash since the airplane was flying too close to the ground to safely reach the airport (Eisen and Savel 2009).

The simplistic representation of some organizational conditions and people’s actions as holes is also exposed to the limitations of hindsight; this is because erroneous actions are only identified after the manifestation of the outcomes. Before the adverse event occurs, it is possible that some holes (risk conditions) are not visible, so without knowledge of the outcome, such conditions are not considered a risky aspect of the system. Only after the accident will investigators, by retracing the event, actually be able to ascertain that there were elements of risk; this revelation occurs because, in hindsight, it becomes evident that these elements indeed contributed to the accident (Dekker 2010). 

In addition, safety, according to the model, derives from solid barriers represented by the slices that contain the risk, and accidents result from the accumulation of multiple failures, each represented as a hole in the cheese slices. However, reality is more complex: An error is not absolute but exists only in relation to other factors. Especially in complex socio-technical systems, accidents may occur not because of holes, but because of dysfunctional interactions among system factors (Hollnagel 2014). Sometimes, the accident may be caused not by a violation, a fault, or a mistake, but it may be due to just a dangerous relationship between two or more factors that, taken in their isolation, are completely safe. What is risky, therefore, is not the hole but its interaction with different organizational conditions. For example, a good procedure may become risky in some conditions, with some operators, and with some tasks. Each of them may be considered healthy and well-performing, but their combination could lead to an accident.

Beyond the biases that could be triggered by the conceptualization of the model as a sequence of solid slices scattered with holes, there is also another drawback: People could go further and come to conclusions that were not in the intentions of the author. A flawed and superficial interpretation of Reason’s model would see the metaphor as a more evolved version of the domino chain of events, priming the observers to simply shift blame from frontline operators to managers, perpetuating the search for a more remote and elusive cause. This could hinder the investigation by focusing on factors that are so general as to be elusive, such as organizational culture. For instance, it is undeniable that the Chernobyl incident can also be interpreted in the context of the political and economic collapse of the Soviet Union, or that the accidents of the Space Shuttle Columbia and Challenger were influenced by the competition between the United States and the USSR in space exploration and NASA’s cost containment policies. However, as Reason (1997) himself has emphasized, perhaps the pendulum has swung too far, shifting from a person-centered approach to a systemic approach where nobody is accountable for the outcomes. There is a risk that interpreting incidents as the sole expression of organizational pathologies compromises a correct holistic view, which should start with individuals and analyze the contexts (Young et al. 2004). Therefore, a true systemic reading does not seek blame in the organization, corporate, or national culture but investigates the conditions at all levels and studies their complex interactions.

#### 2.2.2. How Can the Graphical Representation of Swiss Cheese Model Shape (and Bias) the Observers’ Comprehension of Accidents?

Larouzée (2017) emphasizes the effectiveness of the Swiss cheese model’s graphical representation in facilitating its adoption and dissemination within organizations. This simple yet powerful depiction breaks down a familiar object such as Swiss cheese into slices, enabling observers to grasp the complexity of the model more easily. As a result, it has gained widespread popularity, so that it is considered a common language for understanding accidents among safety professionals (Perneger 2005). 

When the metaphor of Swiss cheese takes on a visual form, it assumes distinct characteristics: In the depiction, slices become physical elements that are very tangible (there are shadows, well-defined outlines, and a sense of depth). In the past, the concept of barriers in the field of safety has been associated with the idea of separating risks from objects or people that could be affected by those risks. According to the perspective of energy and barriers, accidents occur as a result of uncontrolled energy discharge. While some authors have emphasized that barriers can include less directly physical elements, such as alarm systems, many others have preferred to consider barriers in their tangible and physical forms. It is not coincidental that in numerous contexts, the primary actions taken to reduce risk involve physical barriers, such as railings, constrained paths, and hurdles (Rosness et al. 2004). On the conceptual level of the metaphor, slices of cheese represent safety barriers, which are not only physically tangible elements (such as a physical wall separating a moving machine from a worker) but also encompass less tangible aspects such as safety procedures or workers’ actions (Reason 2000). While conceptually these barriers pertain to elements ranging from the physical to the more abstract, their physical concreteness in the Swiss cheese depiction might influence how viewers perceive and interpret them. For instance, a study by Perneger (2005) indicated that participants in the research were able to correctly describe the model and the meaning of the slices but did not recognize safety procedures as barriers because they were perceived as abstract and less concrete than the physical and observable barriers. 

There are other important characteristics of slices of cheese that can guide the comprehension of accidents: Their shape and spatial arrangement. First, in the graphical version, slices take on a defined shape; they are rectangular and uniform in size. However, in everyday experience, the dimensions of slices of cheese vary based on how the cheese is cut, potentially appearing more elongated, flattened, or featuring rounded corners, among other possibilities; these slices can exhibit substantial dissimilarities from one another. Yet, when depicted graphically, all slices are rendered identical, prompting several intriguing questions regarding how observers might perceive this uniformity: Does this uniformity among the slices suggest that safety within organizations is characterized by uniformly standardized safety barriers? If a slice of a distinct shape (e.g., smaller) were depicted compared to another, would it imply that this particular barrier offers lower safety protection? Secondly, as Refaie (2003) argued, the visual communication mode employs spatial visual elements to delineate the temporal dimension, particularly the chronology of events. For instance, it represents elements farther in the past as positioned behind those closer in time. In the Swiss cheese metaphor representation, slices are intentionally depicted one after the other to convey the notion that accidents have a history, which could extend back quite some time. Nonetheless, arranging the slices in this sequential manner raises additional inquiries: Do accidents exclusively occur when this precise alignment of barriers takes place? Considering that accidents happen when holes align, could a safety-improving intervention involve modifying the spatial placement (either higher or lower) of the slices to prevent the alignment of holes with adjacent slices? The perceptions of observers within the graphic representation of this metaphor continue to be uncharted territory in the existing literature. Consequently, significant questions await exploration.

The graphical representation of the model introduces certain elements while not including others from the verbal metaphor. Another effect of the depiction in guiding comprehension of accidents is due to its static nature while aiming to represent highly dynamic phenomena. Reason (1997, p. 9) claimed that the “Swiss cheese” metaphor is best represented by a moving picture, with each defensive layer coming in and out of the frame according to local conductions. Particular defenses can be removed deliberately during calibration, maintenance, and testing, or as the result of errors and violations. Similarly, the holes within each layer could be seen as shifting around, coming and going, shrinking and expanding in response to operator actions and local demands”. Unfortunately, these crucial features could not be inferred by simply looking at the model; practitioners could interpret the metaphor too literally and think about the system levels as static layers (the slices), corrupted by static, and easily recognizable dysfunctions (the holes).

Two significant criticisms have been directed at Reason regarding his metaphorical expression: The utilization of an arrow to symbolize the temporal dimension and the portrayal of latent and active failures. Reason’s graphical representation has faced criticism due to the risk of promoting a mechanistic viewpoint when it comes to comprehending accidents. One of these features is the inclusion of an arrow in the illustration to chronologically depict the steps leading up to the accident. The arrow represents the temporal and spatial alignment of the failures, i.e., their concurrence in the causation of the accident. A misinterpretation of the Reason model might regard this arrow as a mere evolution of the traditional domino chain of events.

Reason always stated that there is an inherent difference between latent failures and active failures; however, they are visually represented with the same shape: The hole in the slice. In the early version of the model (as described by Reason in 1990), a graphical distinction was made between latent failures (managerial), and active failures (unsafe acts). Latent failures were depicted as darker ellipses within rectangles, while active failures were represented as rounder and clearer ellipses. This graphical distinction allowed observers to understand the differences between the two types of failure. However, in more recent graphical iterations of Reason’s model, the visual elements of latent and active failures do not differ (Reason 1997, 2000). 

In conclusion, the Swiss cheese metaphor, as it is visually represented, activates a frame that may bias operators and practitioners in looking for a linear and sequential chain linking visible holes (i.e., faults, errors) at every level of the system. Its effectiveness in analyzing accidents could still be of some value, but it would be very limited in its heuristic power to guide the analysis of complex socio-technical systems before accidents occur.

### 2.3. Complex Non-Linear Models: The Functional Resonance Analysis Method (FRAM)

The evolution of sociotechnical systems and the increase in complexity and interactivity of the elements and functions pushed researchers in safety science to develop new models and methods to tackle the unprecedented challenges. From the 2000s, the literature flourished with proposals that aimed at changing the paradigm towards so-called system resilience by means of complex non-linear models (Le Coze 2022; Saleh et al. 2010). Pillay (2015) defines this era as a “sophisticated” approach since it is based on concepts drawn from the sciences of complexity. New terminology and new theoretical frames of reference are introduced. Resilience is proposed as the organizational cornerstone for safety and is defined as the capacity of a system to maintain its functions before, during, and after unexpected perturbations (Hollnagel et al. 2006). Accidents are considered emergent properties, i.e., events that are generated by the complex interaction of the elements of the system (technological, procedural, human, organizational, physical, etc.). This holistic approach highlights the relationship between functions rather than looking for specific and localized failures. According to this perspective, the single elements may even be considered safe, while the mishap could be due to their interaction.

Hollnagel (2012) uses a metaphor drawn from physics, the concept of resonance, to describe the interaction among functions. In physics, resonance refers to a phenomenon that occurs when an external force or frequency matches the natural frequency of an object or system. When resonance occurs, the object or system vibrates with a significantly increased amplitude, often resulting in enhanced oscillations or vibrations. In structural engineering, resonance can lead to excessive vibrations that can cause damage to buildings or bridges.

These organizational elements resemble vibrating strings, moving back and forth around the optimal operational line. In other words, each function oscillates and has some degree of variability (for instance, operators could follow a procedure more or less strictly). Under specific conditions, the combination of various frequencies and variabilities creates functional resonance, which amplifies the signal, transforming it from weak to strong and eventually leading to an accident. As a result, the occurrence of an adverse event is seen as an emergent property resulting from the intricate interaction and resonance among different components of the system. It is not about direct cause-and-effect relationships, but rather the synergistic effect of diverse elements that greatly magnify the outcome. If the system exhibits strong connections, resonance quickly propagates throughout the entire system and can lead to its collapse.

In addition, rather than representing functions as binary (correct/wrong, functioning/broken, etc.), the resilience approach analyzes the variability of the system’s functions, and rather than investigating the events only when they went bad, it focuses attention also on how and why things usually go well (Dekker 2011; Dekker et al. 2011).

A number of systemic models have been proposed under the framework of resilience and complexity: e.g., the systems theoretic analysis model and processes model (STAMP) (Leveson 2004), the functional resonance analysis method (FRAM) (Hollnagel 2012), and the Accimap (Rasmussen 1997). They all share the same principles, and their visual representations (and metaphors) are usually aimed at depicting the complexity of nodes and connections representing the system’s functions. For the purpose of this analysis, we will focus on the FRAM method, since it is clearly related to a visual representation and is one of the most used by researchers.

The FRAM method moves away from linear cause-and-effect relationships. Instead, it emphasizes the non-linear nature of system behavior, where interactions between functions can have disproportionate effects on outcomes. In addition, the FRAM model aims to enhance system resilience by identifying potential points of vulnerability or failure. It helps in understanding how variations in system functions can either contribute to or mitigate the consequences of adverse events.

Overall, the FRAM method provides a systemic and dynamic perspective on complex systems, emphasizing the interdependencies and interactions between functions, variabilities, and outcomes. It offers a way to analyze and manage system performance with a focus on resilience and the prevention of adverse events.

Hollnagel has repeatedly pointed out that the FRAM is a method rather than a model, even though this distinction is not always respected among researchers since the initial definition of this approach explicitly presents it as a model (Hollnagel and Goteman 2004). The reason may be due to its visual output, which aims at representing the intricate web of relations among the functions. Each function is analyzed from six perspectives:

(a) Input: the starting point of the function; what the function receives to begin its activity.
(b) Output: the result of the function.
(c) Preconditions: conditions that must be in place before the function can be performed.
(d) Resources: what the function needs to have in order to be executed.
(e) Time: time constraints that limit the function.
(f) Control: how the function is monitored and controlled.

The analysis takes into account each function and how its six aspects could have interacted with other functions in order to resonate in an adverse event.

**Figure 4:**
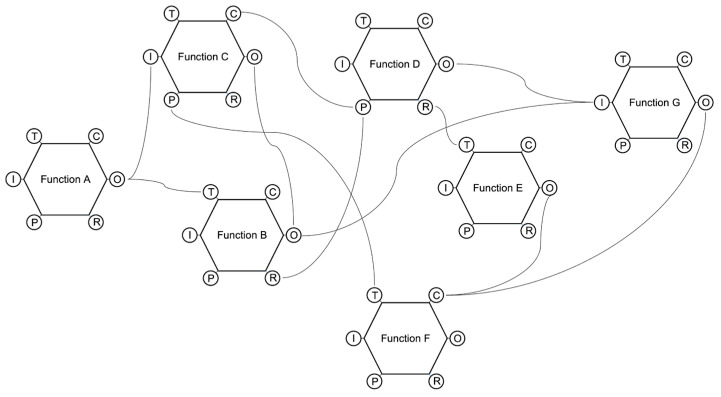
A simplified representation of the visual output of FRAM.

The visual result of FRAM could be quite complex, given the high number of functions analyzed, the numerous interactions among their aspects, and the intricacy of the connections represented as lines linking nodes (see, for instance, O’Hara et al. 2020; Lee et al. 2020) (Figure 4).

#### How Does the Graphical Representation of the FRAM Metaphor Guide the Comprehension of Organizational Accidents?

While the functional resonance analysis method (FRAM) has been generally well-received, there have been some discussions and critiques regarding its complexity (Underwood and Waterson 2012; Dallat et al. 2019; Patriarca et al. 2020). However, it is worth noting that these critiques do not necessarily undermine the method’s overall value and applicability. The conceptual metaphor, related to the physical phenomenon of resonance, is accurate in highlighting the crucial aspects of accident dynamics. However, its graphical representation might show some drawbacks. We will focus here on the critiques concerning the complexity of interpretation of the visual output, leaving behind other critical aspects that are not strictly related to the effects of the visual representation on users’ cognitive processes. 

The model aims at representing complex and non-linear interactions among the system’s functions. Hollnagel did not choose a concrete metaphor for his model; he rather adopted the notion of resonance, trying to convey the idea of accidents as the combination of multiple interactions among functions that follow a non-linear path. This means that small local interactions could result in high resonance on a global scale. The graphical representation is rather abstract and could not help practitioners understand these phenomena. Each function is designed as a hexagon, given its six properties (input, output, preconditions, etc.), and each property could be linked to one or more other properties of one or more other functions. This visual representation guides the analyst in taking into account the fundamental aspects of each function; however, the method does not provide a guide for listing the functions and locating them in the space. As stated by Patriarca et al. (2017), the FRAM is not able to visually reproduce the functional hierarchy of a system (from the global functions to subsystems, to function units and single components). All the functions are represented on a flat surface, and it is not possible to infer their hierarchical relationship. Specifically, “even though FRAM is a well-established method to evaluate non-linear functional interactions among system components, it currently lacks guidance for how to represent large systems, whose descriptions require many interacting and coupled functions. One could argue that this problem could be limited by the differentiation in background and foreground functions, obtaining multiple ‘simple’ models where the background functions of a model are analysed as foreground functions in another one. However, this strategy may cause the lack of a system wide perspective and generate issues in linking different models and understanding the whole complexity. Furthermore, the FRAM functions’ positioning in the space does not have a particular meaning, since the method gives the analyst freedom to place them everywhere, lacking a standard representation” (Patriarca et al. 2017, pp. 10–11).

Another constraint of the FRAM is discussed by Salehi et al. (2021), who argue that it is not helpful in capturing and visualizing the variability of functions, an aspect that is a cornerstone of the approach. The authors propose to enrich the method with the capacity to visualize and understand the qualitative and quantitative characteristics of functional variability. 

In general, one of the main criticisms of FRAM from a graphical point of view is related to its complexity (Rad et al. 2023). The users cannot grasp patterns and have insights looking at the intricate web of connections among dozens of functions, and the visual representation should be decomposed into layers or functional levels in order to facilitate the understanding of the dynamics (Patriarca et al. 2017).

## 3. A Framework for Developing Models of Organizational Accidents

As we have seen, accident causation models are not only tools but rather frames through which we can look at organizational dynamics. Our reflection upon models should now take into account three main actors: safety scholars (who develop more and more sophisticated models), safety practitioners (who need to adopt the models to understand accidents and promote safety in their workplace), and workers (who daily cope with operational challenges at the “front-line”, and perceive and manage risks thanks to the cultural frames shared in their workplace).

The flourishing of theories and models of the last decades has not been followed by an update of safety practitioners’ methods for accident investigation and analysis. While scholars tried to develop accurate and comprehensive models and metaphors to capture the challenging nature of complex socio-technical systems, safety practitioners were reluctant to adopt new frames for investigating organizational accidents. According to Leveson (2011), the linear cause–effect model is predominant in accident reports, and the line of inquiry usually stops too early in the supposed chain of responsibility. Systemic models, although scientifically sound, seem to be less preferred by professionals, which refer to the sequential representation of accidents still available in the practitioner-focused literature proposed by national safety institutions and safety boards (Okstad et al. 2012; Roelen et al. 2011; Salmon et al. 2012). In particular, in some operational domains, the Swiss cheese model may still be considered the cutting edge of accident modeling (Roelen et al. 2011). On the other hand, even when practitioners claim to know more sophisticated models, they are considered too difficult to apply because they require a considerable amount of theoretical background and are usually much more time consuming to learn and apply (Ferjencik 2011; Johansson and Lindgren 2008; Underwood and Waterson 2013, 2014). 

The gap between research and practice seems wide, and the proposal of new and more sophisticated models could be useless if we do not understand the reasons behind this neglect by practitioners. In an interesting study to investigate this gap, Underwood and Waterson (2013) compared safety practitioners and researchers against their awareness, adoption, and usage of five accident causation models (from linear to systemic): Fault tree, Swiss cheese, Accimap, STAMP, and FRAM. The differences in model awareness were remarkable, in particular concerning the systemic non-linear models (Accimap, STAMP, and FRAM), which were neglected by more than 60% of the practitioners. Notwithstanding that researchers generally have some awareness of sophisticated models, both they and the practitioners declare a preponderant adoption of the fault tree model (related to the domino model) and the Swiss cheese model. The lack of awareness may be due to the sources of information that, for the systemic and more advanced models, are scientific conferences and peer-reviewed journals. Practitioners do not primarily update their knowledge and working methods through the sources used by researchers. In addition, the adoption of methods is mainly based on their usability (54% of responses), while their validity was mentioned only by 27% of respondents. Therefore, if the model is considered too conceptual, difficult to understand, or hard to apply, the practitioners will prefer a well-known method. Moreover, if accountability or blame drives the inquiry, more linear models could be preferred since they are quicker and easier to find the scapegoat or weak link in the chain. Other reasons could hinder the usage of systemic models, such as access to subject matter experts, the need for resources to carry on investigations at a deeper level, access to considerable amounts of data or access to sources that are not easy to inquire about, and the organizational safety culture.

If “sophisticated” models such as FRAM, albeit accurate and more tuned for systemic dynamics, are not adopted in favor of more usable and understandable linear models, we expect that safety practitioners may still be reading their work environment with those linear frames of reference. 

If we notice a gap between scholars and practitioners in the most favored models, the literature is completely oblivious to the third kind of stakeholders we mentioned before: Workers. It is not clear how the models adopted by safety practitioners can influence the perception of risks and the behavior of front-line operators. We believe this line of research deserves further investigation, and we hypothesize that a shared metaphor among safety practitioners and workers should be necessary to develop a safety culture.

Do we need a new model? We could say “no” and “yes”. Namely, we do not need new accident models since the sophistication and theoretical rigor of the currently available models are undisputable. But we need models that could maintain the systemic nature of the non-linear approaches while overcoming their usability limitations and graphical effectiveness. 

Following Hovden et al. (2010) suggestion, any new safety model and metaphor should be based on the following criteria:

1. create the ground for a shared interpretation of accidents through a simplified description of the relevant phenomena;
2. provide a tool for framing and communicating safety issues to all levels of an organization;
3. enable people to analyze the accident, preventing personal biases and opening the door for effective solutions;
4. guide investigations in terms of which data to collect and analyze and how to process them;
5. highlight and facilitate the analysis of interactions between factors and conditions behind an accident.

In other words, the above-mentioned criteria pertain to the model’s effectiveness in facilitating the analysis, the communication, and the investigation of a safety-relevant event. 

Since the models and metaphors should represent complex dynamics in socio-technical systems, they should be able to facilitate practitioners in developing system thinking in accident analysis. The visual features of the models and metaphors are therefore important since they guide (and sometimes bias) the reflection on organizational accidents and risk assessment. 

In Table 1, we list some tenets to guide the design of metaphors aimed at facilitating system thinking in accident analysis (Rad et al. 2023; Underwood and Waterson 2014).

## 4. Conclusions

More research is needed on the relationship between the visual features of a model and the cognitive processes that are promoted by its graphical characteristics, and even the conceptual metaphor that is chosen to describe organizational accidents. In this paper, we reported the current evidence concerning possible effects, biases, and even misunderstandings based on the accident metaphors used for three paradigmatic accident causation models: The domino model, the Swiss cheese model, and FRAM. They represent an interesting abstraction progression: The domino model literally represents the linear chain reaction of causes and effects; the Swiss cheese model is based on a concrete metaphor (the slices of cheese and the holes), but requires the user to have a bit more abstraction in representing hole movements and the concept of alignment. Finally, the FRAM does not use any concrete metaphor; rather, it tries to visualize the physics concept of resonance linking the edges of hexagons, representing the system’s functions. We addressed issues concerning these models, guided by two questions: How do metaphors influence the comprehension of organizational accidents? And how can the graphical representation of metaphors shape the observers’ comprehension of accidents? Specifically, the domino model metaphor in itself, more than its graphical representation, can guide (and bias) cognition about organizational accidents. The Swiss cheese model shows drawbacks both as a metaphor and concerning the way it has been visually represented. The FRAM model, on the other hand, is a solid metaphor whose graphical representation has some criticalities.

We may say that the more ambitious and sophisticated the models, the less concrete their metaphor is. This impoverishment in the visual component may be due to the very nature of the phenomena they aim to represent, which are less concrete than tools, people, and physical spaces, and are more abstract, such as interactions, functions, emergent properties, etc. However, we argue that this drift towards the abstraction of metaphors and impoverishment of visual representations may have at least two drawbacks. First, the effectiveness of metaphors is weakened since the destination domain (i.e., the ineffable complexity of modern systems) cannot be understood thanks to the link with a familiar source domain (the visual features of the model) if the source domain is too abstract and complex in itself. Second, the models must be used by practitioners, and if they are too complex to use and communicate, they lose effectiveness. 

The choice of a specific metaphor is a positioning of the authors about the phenomenon they want to describe. It tells how they look at the phenomenon, what they want to highlight, and what they discard as irrelevant. Metaphors cannot represent all the characteristics of a phenomenon, just like a map does not represent all the territory. It is a matter of choice. Therefore, the problem is not that accident metaphors reduce the complexity of the reality they want to describe, but that this reduction does not discard essential aspects and does not bias cognition about safety issues. The challenge for scholars is to choose a metaphor that is able to highlight the relevant dynamics of organizational accidents while neglecting irrelevant aspects. The metaphor becomes the tool guiding the comprehension and management of safety in complex socio-technical systems. In this paper, we argue that an informed choice should take into account not only the metaphor per se but also its graphical representation. 

Researchers lead the models towards a good level of accuracy, while safety practitioners working in organizations pull towards usability. Nonetheless, if we consider accident causation models not only as a good tool to analyze an accident but also as a framework to promote safety and foster prevention, we need to introduce a third party: The people in the organization that are not technically in charge of analyzing and investigating accidents but are the real actors of safety. They define strategies, apply procedures, organize environments, adapt rules to contingent situations, notice potential threats, make decisions, plan actions, and interact with automation and other people inside and outside the system. All these actions and thoughts should take place within a common framework, representing system dynamics and helping people to be proactive and sensitive to risks.

Complexity is studied by researchers, accidents are investigated by risk managers, and safety is conducted by people within the organization in their everyday actions. We believe that one of the reasons for the Swiss cheese model’s worldwide success is that it was easy enough to be used as a framework for every worker in the system and usable enough to represent the course of events. However, systems’ complexity has grown, and, as claimed by resilience scholars, today we need models that can tackle the non-linearity of systems, an aspect that is not very well represented by Reason’s model.

Therefore, we claim that a good model should balance the three characteristics of being accurate in representing systemic dynamics, being usable for safety analysts, and being effective for sharing safety messages within the system. The challenge for a metaphor is to satisfy these three aspects at the same time.

Safety is an emergent property of a system where the most flexible elements are the people operating within and around it. If they can share a common perspective on a system’s dynamics and can understand how accidents occurred in the past, they can learn from experience and foster resilience.

## Figures and Tables

**Table 1:** A framework for organizational accidents model design.

Feature	Rationale	Model’s Functions
System structure	Complex systems are often hierarchically organized	The model should be able to: represent the different levels of system represent their relationships highlighting the different functions (from the goals of the organizational level, to the functions of the “sharp end”) represent the boundaries of the system, distinguishing it from its environment
System components relationships	Accidents are emergent properties of the system several components	The model should be able to: promote a holistic approach to the analysis represent the several components represent components’ relationships integrate technical, organizational, and human factors represent their inter-relations and intra-relations
System behavior	Socio-technical systems dynamically balance production and protection	The model should be able to: represent equifinality (where a given end state can be achieved by means of multiple starting points) represent multifinality (a given starting point can produce several outputs); represent the dynamic adaptations to internal and external perturbations represent their position in relation to the safety boundary
